# KNNCNV: A K-Nearest Neighbor Based Method for Detection of Copy Number Variations Using NGS Data

**DOI:** 10.3389/fcell.2021.796249

**Published:** 2021-12-22

**Authors:** Kun Xie, Kang Liu, Haque A K Alvi, Yuehui Chen, Shuzhen Wang, Xiguo Yuan

**Affiliations:** ^1^ School of Computer Science and Technology, Xidian University, Xi’an, China; ^2^ Hangzhou Institute of Technology, Xidian University, Hangzhou, China; ^3^ Shandong Provincial Key Laboratory of Network Based Intelligent Computing, University of Jinan, Jinan, China

**Keywords:** k-nearest neighbor, copy number variation, next-generation sequencing, variational Bayesian Gaussian mixture model, tumor genome

## Abstract

Copy number variation (CNV) is a well-known type of genomic mutation that is associated with the development of human cancer diseases. Detection of CNVs from the human genome is a crucial step for the pipeline of starting from mutation analysis to cancer disease diagnosis and treatment. Next-generation sequencing (NGS) data provides an unprecedented opportunity for CNVs detection at the base-level resolution, and currently, many methods have been developed for CNVs detection using NGS data. However, due to the intrinsic complexity of CNVs structures and NGS data itself, accurate detection of CNVs still faces many challenges. In this paper, we present an alternative method, called KNNCNV (K-Nearest Neighbor based CNV detection), for the detection of CNVs using NGS data. Compared to current methods, KNNCNV has several distinctive features: 1) it assigns an outlier score to each genome segment based solely on its first *k* nearest-neighbor distances, which is not only easy to extend to other data types but also improves the power of discovering CNVs, especially the local CNVs that are likely to be masked by their surrounding regions; 2) it employs the variational Bayesian Gaussian mixture model (VBGMM) to transform these scores into a series of binary labels without a user-defined threshold. To evaluate the performance of KNNCNV, we conduct both simulation and real sequencing data experiments and make comparisons with peer methods. The experimental results show that KNNCNV could derive better performance than others in terms of F1-score.

## Introduction

Copy number variations (CNVs) of DNA sequences are accountable for functional phenotypic diversity in many species and play an important role in human genomic variation and cancer initiation (Schrider et al., 2013; Unckless et al., 2016). CNV is a commonly reported variation from the diploid state caused by amplification or deletion of genomic regions ranging from one kilo-base to several mega-bases (Redon et al., 2006; Li et al., 2020). In cancer, tumor-derived CNVs are one of the most significant genomic anomalies, alongside somatic mutations and structural variations (SVs). Tumor suppressor gene inactivation or oncogene activation are frequently ascribed to copy number loss or gain, respectively (Yuan et al., 2012). Specifically, Gains may contain oncogenes, and losses may include tumor-suppressor genes (Xie et al., 2021). Consequently, detecting cancer-associated copy number occurrences is crucial in identifying patient subtypes, as well as providing insights into prognosis and prospective treatment options. Fortunately, next-generation sequencing (NGS) technology has accelerated the development of the detection of CNVs (Teo et al., 2012), which provides greater scope to discover novel CNVs and has a greater resolution to forecast both breakpoints and shorter CNVs. However, owing to the intrinsic complexity of CNVs structure and the huge scale of NGS data, accurate detection of CNVs remains challenging.

Numerous bioinformatics tools for the detection of CNVs from NGS data have been developed, and these algorithms can be classified into four main categories: read-pair (RP), split-read (SR), read-depth (RD), and *de novo* assembly (DA). The above four approaches have their strengths, shortcomings, and scope of implementation, and their details can be referred to (Zhao et al., 2013; K. Ye et al., 2016). Among these approaches, the RD-based strategy is most frequently used to detect CNVs, since the strategy is theoretically more likely to detect CNVs with different sizes (Zare et al., 2017). A great number of methods under the RD-based strategy have been developed based on the characteristics of NGS data. FREEC (Boeva et al., 2010; Boeva et al., 2012) considers the RD profile from a global context and exploits the variance in RD values to discover CNVs. When normal matched samples are not present, FREEC can use GC-content to normalize the RD values and accurately identify CNVs from tumor samples. ReadDepth (Miller et al., 2011) and iCopyDAV (Dharanipragada et al., 2018) are similar approaches. The *m*-HMM (Wang et al., 2014) method considers the entire RD profile as a Markov model and forecasts copy number states. CNVnator (Abyzov et al., 2011) leverages the multiple-bandwidth partitioning technique and mean-shift approach to detect broad CNVs. GROM-RD (Smith et al., 2015) can analyze multiple biases such as GC-bias and repeat bias and use sliding windows with variable size to improve breakpoint resolution.

The above methods take different perspectives on the features of CNVs, and such methods have the advantage of detecting broad CNVs. However, the focal (*i.e.*, local) CNVs may be ignored. To address the above limitation, the CNV-LOF (Yuan et al., 2021a) takes a local view on the RD values, so the method avoids some local CNVs being masked by the surrounding regions. To consider the correlation of copy numbers in adjacent positions, CNV_IFTV (Yuan et al., 2021b) calculates outlier scores based upon the isolation forest algorithm and leverages the total variation model to smooth these scores, and similar methods include CNV-RF (Onsongo et al., 2016) and CONDEL (Yuan et al., 2020). In addition, IhbyCNV (Xie et al., 2021) takes a comprehensive viewpoint on the characteristics of CNVs, that is, the method treats CNVs detection as outlier events from five perspectives on the RD profile to be addressed. Although these methods exhibit their own characteristics and advantages in different scenarios, it is still necessary to design a simple and effective method to deal with the intrinsic complexity of CNVs structure and NGS data itself.

With careful consideration of the challenges above, in this paper, we propose an alternative method used for whole genome sequencing, coined KNNCNV (K-Nearest Neighbor based CNV detection), which can identify CNVs using NGS data. The core module of the KNNCNV is that the outlier scores for all genome segments are calculated solely by their *k*th nearest-neighbor distances, and then these scores are converted into a succession of binary labels through the VBGMM (Corduneanu and Bishop, 2001; Tzikas et al., 2008). In this work, we make two key contributions as follows.

1) The outlier score for any genome segment can be defined based solely on its first *k* nearest-neighbor distances. More specifically, the average value of these distances is regarded as the outlier score of the genome segment, which is not only easy to extend to other data types but also boosts the power of detection CNVs, especially the local CNVs that are likely to be masked by their surrounding regions.
2) This paper leverages the VBGMM to convert the outlier scores for all genome segments into a series of binary labels that can indicate which genome segments are CNVs. The VBGMM can approximate the posterior distribution of these scores, so it can also be considered as a soft clustering method, and these binary labels are obtained without a pre-specified threshold.

## Materials and Methods

### Overview of KNNCNV

**FIGURE 1:**
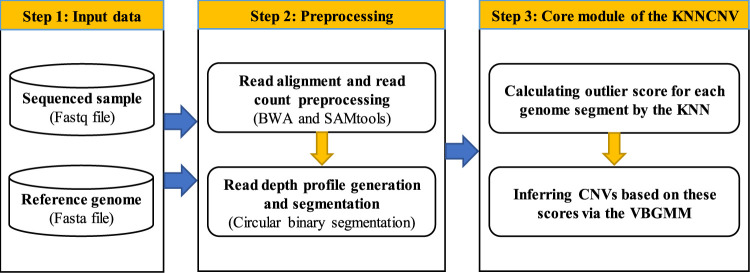
Workflow of the KNNCNV method.

The workflow of the KNNCNV method is shown in Figure 1, which consists mainly of three steps. In the first step, a sequenced sample and a reference genome are taken as the input data. The second step is preprocessing, including the read alignment, read count (RC) preprocessing, and read depth (RD) profile generation and segmentation. In the third step, the outlier score for each genome segment is calculated by the *k*-nearest neighbor (KNN) (Ramaswamy et al., 2000; Angiulli and Pizzuti, 2002), and these scores are converted into binary labels via the VBGMM (Corduneanu and Bishop, 2001; Tzikas et al., 2008). In addition, the KNNCNV is implemented in Python and R language, which is freely available at https://github.com/BDanalysis/KNNCNV.

### Preprocessing

After obtaining a sequenced sample (*i.e.*, a Fastq file) and a reference genome (*i.e.*, a Fasta file), the sequenced sample is aligned to the reference genome with the BWA algorithm (Li and Durbin, 2010). Then the alignment result is extracted by the SAMtools software (Li et al., 2009), and the RC profile, which is SAM or BAM format, is obtained. The preprocessing of the RC profile includes the preprocessing of the reference genome, generating the genome bins, and correcting the GC-bias. The reference genome has some problems with missing positions and ‘N’ positions. In this paper, the missing positions are filled with zeros, and the ‘N’ positions are removed. As for generating the genome bins, the RC profile is partitioned into continuous and disjoint genome bins with the same length *L*_*b*_ (*i.e.*, the bin size *L*_*b*_ equals 1,000 bp). The average RC value for each genome bin is regarded as its RD value, and simultaneously the fraction of GC-content can be obtained. In terms of the GC-bias, it is corrected by the prior work (Yuan et al., 2021a). Owing to the correlations between adjacent bins (Yuan et al., 2018; Yuan et al., 2021a), segment-based units have some advantages over bin-based ones in both computational cost and reliable results. Therefore, the whole genome is divided into continuous and non-overlapping regions with the same length *L*_*r*_ (*i.e.*, the region size *L*_*r*_ is equal to 50,000 bp), and then each region is segmented by the circular binary segmentation (CBS) algorithm (Venkatraman and Olshen, 2007). Thus, a family of genome segments that are different in size are generated in each region, and the number of genome segments relies on the fluctuation of the RD values. Let the number of all genome segments generated by all regions be *N*, hence all genome segments are denoted as $R={\left[r_{1},r_{2},\cdots ,r_{N}\right]}^{T}\in R^{N\times 1}$ , where $r_{i}$ represents average RD values for all genome bins in the *i*th genome segment, and ${\left[\cdot \right]}^{T}$ is a transposed matrix.

### Calculating Outlier Score for Each Genome Segment by the KNN

After the above preprocessing, the RD values for all genome segments (*i.e.*, *R*) can be obtained. Next, to estimate the degree of abnormality (*i.e.*, outlier score) of each genome segment, we resort to the *k*-nearest neighbor (KNN) method (Ramaswamy et al., 2000; Angiulli and Pizzuti, 2002), which is a distance-based method and naturally assumes that the *k*-nearest neighbor distance of outliers (*i.e.*, CNVs) is much larger than that of normal points. To simplify the representation, we use *k* to denote the number of nearest neighbors for any object. Before the introduction of calculating the outlier score by the KNN, we first describe two definitions. Note that Definitions 1 refers to prior work (Breunig et al., 2000; Yuan et al., 2021a), and similarly, Definition 2 refers to (Ramaswamy et al., 2000; Angiulli and Pizzuti, 2002; Aggarwal, 2017).

Definition 1 (*k*-*distance* and *k*-nearest neighborhood for any object *r*) Given the RD values for all genome segments $R={\left[r_{1},r_{2},\cdots ,r_{N}\right]}^{T}\in R^{N\times 1}$ and a positive integer *k*, the *k-distance* for any r∈R to the remaining ones can be defined as k-distance(r)=distance(r,e) , where e∈R , and *distance* (*r*, *e*) denotes the Euclidean distance between objects *r* and *e*. Moreover, among all objects in *R*, *e* is an object that is the *k*th nearest neighbor to *r*. The *k*-nearest neighborhood for any object r∈R can be formulated as $N_{k}\left(r\right)=\left\{t|distance\left(r,t\right)\leq k-distance\left(r\right),t\in R,t\neq r\right\}$ . Thus, the first *k* nearest-neighbor distances between the object *r* and the rest can be expressed as $\left\{distance\left(r,t\right)|t\in N_{k}\left(r\right)\right\}$ .

Definition 2 (outlier score for any object *r*) Knowing the RD values for all genome segments $R={\left[r_{1},r_{2},\cdots ,r_{N}\right]}^{T}\in R^{N\times 1}$ and a positive integer *k*, the outlier score for any object r∈R can be defined as 1|Nk(r)|∑t∈Nk(r)distance(r,t) , where $\left|N_{k}\left(r\right)\right|$ denotes the cardinality of the set $N_{k}\left(r\right)$ , and $0\leq \left|N_{k}\left(r\right)\right|\leq k$ . Furthermore, see Definitions 1 for more information on distance(r,t) and $N_{k}\left(r\right)$ . Note that the above scheme for calculating outlier scores is referred to as the average outlier score scheme.

From the above definitions, it is obvious that Euclidean distances between all pairwise objects must be calculated to obtain the *k*-nearest neighborhood for all objects in *R*. The computational overhead $O\left(N^{2}\right)$ increases significantly with the increase of *N*, where *N* denotes the number of genome segments. To partially circumvent this problem, a space-partitioning tree data structure, *k*-dimensional tree (KDTree) (Ramasubramanian and Paliwal, 1992), is adopted to search the *k*-nearest neighborhood for any objects in *R*. On utilizing the KDTree, its computational cost is O(N⁡log⁡N) . Next, this paper introduces how to estimate the outlier score for each genome segment via the KNN method. Given the RD values for all genome segments $R={\left[r_{1},r_{2},\cdots ,r_{N}\right]}^{T}\in R^{N\times 1}$ and a positive integer *k*, the outlier score $s_{r}$ for any object r∈R can be obtained by Definition 2. More exactly, the score $s_{r}$ is defined as the average value among its first *k* nearest-neighbor distances. Additionally, there are two simple variations of the scoring mechanism corresponding to the largest outlier score scheme and the median outlier score scheme (Aggarwal, 2017). Precisely, for any object r∈R , the two simple variations treat the largest and median value among the first *k* nearest-neighbor distances as its outlier score, respectively. Nevertheless, the two simple variations neglect or hardly consider the information of other nearest neighbors, which may yield unstable performance when the *k* value is not reasonable. Therefore, this paper adopts a more robust average outlier score scheme to estimate the outlier score for any object in *R*. It is noteworthy that, among these three outlier score schemes, a ‘correct’ *k* value should be specified in advance (Aggarwal, 2017). However, in the detection of CNVs, it is difficult to search for a ‘correct’ *k* value due to the lack of ground truth. To partially bypass this issue, one specifies a range of values of *k* and then leverages random strategy to determine a final *k* value. More specifically, the integer *k* is randomly selected in the range of [0.2N,0.35N] , and *k* is rounded down. Accordingly, outlier scores for all genome segments (*i.e.*, $S={\left[s_{1},s_{2},\cdots ,s_{N}\right]}^{T}\in R^{N\times 1}$ ) are obtained through the average outlier score scheme, as shown in Definition 2.

### Inferring CNVs Based on the Scores via the VBGMM

Although outlier scores for all genome segments are obtained, these scores cannot be directly used to determine which genome segments are CNVs. A simple solution is that after the outlier scores are ranked in descending order, the solution treats the genome segments corresponding to the first *n* scores as CNVs, and the solution is called a simple threshold scheme in this paper. Although converting these scores into binary labels is feasible by the simple threshold, it is difficult to define a reasonable *n* value owing to the absence of ground truth. To address this issue, the variational Bayesian Gaussian mixture model (VBGMM) (Corduneanu and Bishop, 2001; Tzikas et al., 2008) is adopted to convert the outlier scores into a series of binary labels. The VBGMM can approximate the posterior distribution of these scores, so the method can also be considered as a soft clustering method, and these labels are obtained without a user-defined threshold.

This paper first introduces the Gaussian mixture model (GMM) (Bishop, 2007), which assumes that the distribution of *S* can be represented by the linear superposition of *M* Gaussian distributions (*i.e.*, component). Let $\alpha_{m}$ and $N\left(S|\mu_{m},\sigma_{m}^{2}\right)$ be the mixing coefficient and the probability density of the *m*th component, respectively, where $\mu_{m}$ and $\sigma_{m}^{2}$ represent the mean and variance. Note that we use μ to denote the set $\left\{\mu_{1},\mu_{2},\cdots ,\mu_{M}\right\}$ , and similarly for $\sigma^{2}=\left\{\sigma_{1}^{2},\sigma_{2}^{2},\cdots ,\sigma_{M}^{2}\right\}$ and $\alpha =\left\{\alpha_{1},\alpha_{2},\cdots ,\alpha_{M}\right\}$ . Thus, the mixture distribution of the *i*th outlier score $s_{i}$ can be formulated as Eq. 1.

$$p\left(s_{i}|\alpha ,\mu ,\sigma^{2}\right)=\sum_{m=1}^{M}\alpha_{m}N\left(s_{i}|\mu_{m},\sigma_{m}^{2}\right).$$ (1)

The left and right sides of Eq. 1 integrate $s_{i}$ at the same time. $0\leq \alpha_{m}\leq 1$ and $\sum_{m=1}^{M}\alpha_{m}=1$ are obtained due to $p\left(s_{i}|\alpha ,\mu ,\sigma^{2}\right)\geq 0$ and $N\left(s_{i}|\mu_{m},\sigma_{m}^{2}\right)\geq 0$ . To calculate the parameters of μ , $\sigma^{2}$ , and α , binary latent variables are introduced, which can be defined as $Z=\{z_{im}|1\leq i\leq N,1\leq m\leq M,z_{im}\in \left\{0,1\right\}\}$ , where $\sum_{m=1}^{M}z_{im}=1$ , and $z_{im}=1$ means that $s_{i}$ is sampled from the *m*th component. Therefore, the marginal distribution of *Z* can be formulated as Eq. 2.

$$p\left(Z|\alpha \right)=\prod_{i=1}^{N}\prod_{m=1}^{M}\alpha_{m}^{z_{im}}.$$ (2)

To simplify some representations, let θ be $\left\{Z,\mu ,\sigma^{2}\right\}$ . Given the parameters θ , the conditional probability of *S* can be formulated as Eq. 3.

$$p\left(S|\theta \right)=p\left(S|Z,\mu ,\sigma^{2}\right)=\prod_{i=1}^{N}\prod_{m=1}^{M}N{\left(s_{i}|\mu_{m},\sigma_{m}^{2}\right)}^{z_{im}}.$$ (3)

According to Bayes’ theorem, after the $s_{i}$ is observed, the posterior distribution $p\left(z_{im}=1|s_{i}\right)$ from the *m*th component can be formulated as Eq. 4.

$$p\left(z_{im}=1|s_{i}\right)=\frac{p\left(s_{i}|z_{im}=1\right)p\left(z_{im}=1\right)}{p\left(s_{i}\right)}=\frac{\alpha_{m}N\left(s_{i}|\mu_{m},\sigma_{m}^{2}\right)}{\sum_{m=1}^{M}\alpha_{m}N\left(s_{i}|\mu_{m},\sigma_{m}^{2}\right)},$$ (4)

where $p\left(z_{im}=1|s_{i}\right)$ is also referred to as the responsibility $\gamma \left(z_{im}\right)$ of the *m*th component to $s_{i}$ , that is, $\gamma \left(z_{im}\right)=p\left(z_{im}=1|s_{i}\right)$ . Given the mixing coefficients and the parameters of components, the likelihood function can be formulated as Eq. 5.

$$p\left(S|\alpha ,\mu ,\sigma^{2}\right)=\prod_{i=1}^{N}\left[\sum_{m=1}^{M}\alpha_{m}N\left(s_{i}|\mu_{m},\sigma_{m}^{2}\right)\right].$$ (5)

On obtaining the likelihood function, the parameters of the GMM can be estimated by using the maximum likelihood framework of expectation maximization (EM) algorithm (Zandi et al., 2013). However, the likelihood function may lead to singularities, that is, one or more component density collapses onto specific data (Bishop, 2007). Therefore, this paper utilizes the VBGMM to infer CNVs based on the outlier scores for all genome segments. Precisely, the VBGMM uses a simpler distribution q(θ) to estimate the true posterior distribution p(θ|S,α) and then maximizes the evidence lower bound (ELOB) on ln⁡p(S|α) .

Next, the details of the VBGMM are described in the following. By Bayes’ theorem, we have:

$$\begin{array}{c} \ln p\left(S|\alpha \right)=\ln p\left(S,\theta \text{|}\alpha \right)-\ln p\left(\theta |S,\alpha \right)\\ =\left[\ln p\left(S,\theta \text{|}\alpha \right)-\ln q\left(\theta \right)\right]-\left[\ln p\left(\theta |S,\alpha \right)-\ln q\left(\theta \right)\right]\\ =\ln\frac{p\left(S,\theta \text{|}\alpha \right)}{q\left(\theta \right)}-\ln\frac{p\left(\theta |S,\alpha \right)}{q\left(\theta \right)}, \end{array}$$ (6)

the left and right of Eq. 6 calculate the expectation to q(θ) at the same time, thus Eq. 7 is obtained.

$$\begin{array}{c} \ln p\left(S|\alpha \right)=\underset{L\left(q\right)}{\underset{︸}{\int q\left(\theta \right)\ln\frac{p\left(S,\theta \text{|}\alpha \right)}{q\left(\theta \right)}d\theta }}\underset{KL\left(q|p\right)}{\underset{︸}{-\int q\left(\theta \right)\ln\frac{p\left(\theta |S,\alpha \right)}{q\left(\theta \right)}d\theta }}\\ =L\left(q\right)+KL\left(q|p\right), \end{array}$$ (7)

where L(q) denotes the ELOB on ln⁡p(S|α) , and KL(q|p) denotes the Kullback-Leibler divergence between q(θ) and p(θ|S,α) . Since KL(q|p)≥0 , the ELOB L(q) is less than or equal to ln⁡p(S|α) . The goal of the VBGMM is to select a reasonable q(θ) to approximate the true posterior distribution p(θ|S,α) , that is, minimization KL(q|p) . Of course, the ideal state is KL(q|p)=0 , in other words, q(θ)=p(θ|S,α) and ln⁡p(S|α)=L(q) . Additionally, the ln⁡p(S|α) is fixed relative to the selection of q(θ) , so minimizing the KL(q|p) is equivalent to maximizing the ELOB L(q) . To simplify this problem, it is assumed that the q(θ) follows the mean field theory (Bishop, 2007). Accordingly, the q(θ) can be formulated as $q\left(\theta \right)=\prod_{j}q_{j}\left(\theta_{j}\right)=q_{Z}\left(Z\right)q_{\mu }\left(\mu \right)q_{\sigma^{2}}\left(\sigma^{2}\right)$ . By maximizing the ELOB on ln⁡p(S|α) , the solution of variational posterior $q_{j}\left(\theta_{j}\right)$ can be formulated as Eq. 8.

$$q_{j}\left(\theta_{j}\right)=\frac{\exp E_{l\neq j}\left[\ln p\left(S,\theta |\alpha \right)\right]}{\int \exp E_{l\neq j}\left[\ln p\left(S,\theta |\alpha \right)\right]d\theta_{j}},$$ (8)

where $E_{l\neq j}\left[\cdot \right]$ represents the expectations with respect to $q_{l}\left(\theta_{l}\right)$ for all l≠j . Refer to prior works (Corduneanu and Bishop, 2001; Tzikas et al., 2008) for the specific derivation process. In addition, according to previous work (Corduneanu and Bishop, 2001), the joint distribution p(S,θ|α) can be formulated as Eq. 9.

$$p\left(S,\theta |\alpha \right)=p\left(S,Z,\mu ,\sigma^{2}|\alpha \right)=p\left(S|Z,\mu ,\sigma^{2}\right)p\left(Z|\alpha \right)p\left(\mu \right)p\left(\sigma^{2}\right),$$ (9)

where p(μ) and $p\left(\sigma^{2}\right)$ follow the Gaussian distribution and the Wishart distribution, respectively. Their specific forms refer to (Corduneanu and Bishop, 2001). Thus, considering Eqs. 2, 3, 8, 9 jointly, the iterative formula of the variational posterior $q_{j}\left(\theta_{j}\right)$ can be formulated as Eq. 10.

$$\begin{array}{l} q_{Z}(Z)=\prod_{i=1}^{N}\prod_{m=1}^{M}h_{im}^{z_{im}}\\ q_{\mu }(\mu )=\prod_{m=1}^{M}N(\mu_{m}|b_{\mu }^{m},\sigma_{m}^{2}(\mu ))\\ q_{\sigma^{2}}(\sigma^{2})=\prod_{m=1}^{M}W(\sigma_{m}^{2}|v_{\sigma^{2}}^{\left(m\right)},V_{\sigma^{2}}^{\left(m\right)}) \end{array},$$ (10)

where W represents the Wishart distributions. For more information on $h_{im}$ , $b_{\mu }^{m}$ , $\sigma_{m}^{2}\left(\mu \right)$ , $v_{\sigma^{2}}^{\left(m\right)}$ , and $V_{\sigma^{2}}^{\left(m\right)}$ , please refer to (Corduneanu and Bishop, 2001). After obtaining these variational posteriors, the ELOB L(q) is also obtained. Next, let the partial derivative of ELOB L(q) with respect to α be zero, so the iterative formula of α can be formulated as Eq. 11.

$$\alpha_{m}=\frac{1}{N}\sum_{i=1}^{N}h_{im},$$ (11)

note that details on $h_{im}$ can be obtained by referring to previous work (Corduneanu and Bishop, 2001). The maximum likelihood framework of the EM algorithm is summarized as the following two steps. In the expectation step, the solutions of variational posterior $q_{j}\left(\theta_{j}\right)$ are calculated by Eq. 10. In the maximization step, the iterative formula of α is obtained by maximizing the ELOB L(q) with respect to α . Repeat the above expectation and maximization steps until the stop condition is met (*e.g.*, the maximum number of iterations is reached).

On the basis of the above introduction, one can find a q(θ) to approximate the true posterior distribution p(θ|S,α) . Thus, the outlier scores *S* can be composed of *M* clusters, and each cluster corresponds to a component. The cluster index $\lambda_{i}$ for any score $s_{i}$ can be defined as Eq. 12.

$$\lambda_{i}=\arg\max\left\{\gamma_{im}|1\leq m\leq M\right\},$$ (12)

where argmax{⋅} denotes the index corresponding to the maximum value in the set {⋅} . Consequently, the VBGMM can be regarded as a soft clustering method (Tzikas et al., 2008). Since the outlier scores indicate the anomaly degree of each genome segment, each segment is either a CNV or a normal one. Thus, let *M* equal two, that is, it is assumed that the distribution of *S* can be represented by the linear superposition of two Gaussian distributions. Note that the VBGMM is implemented by scikit-learn (Pedregosa et al., 2011), and the detailed architecture of the VBGMM is described in **Algorithm 1**.

**Algorithm 1: Converting outlier scores into binary labels by the VBGMM**


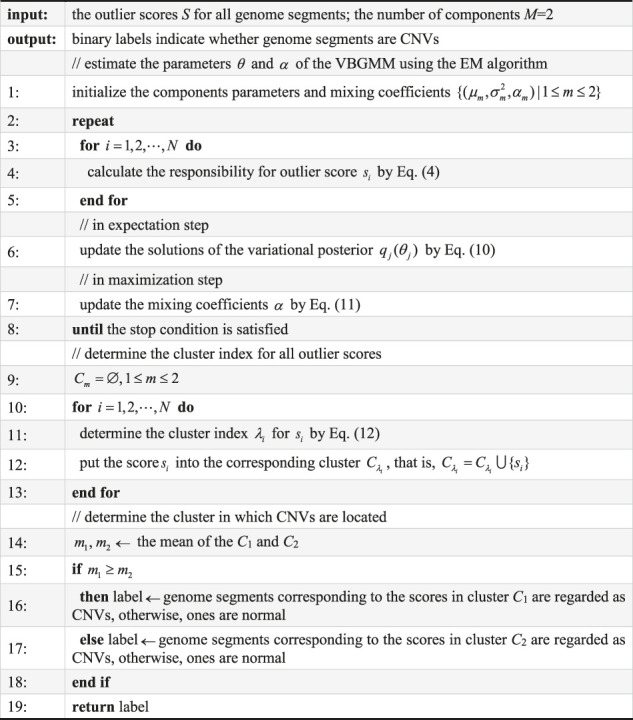


## Results

To evaluate the performance of KNNCNV, we conduct experiments on simulated and real datasets. As for the experiments on the simulated datasets and real blood datasets, we first make comparisons between the proposed method and peer methods and then discuss the influence of the hyperparameter *k* on the result of KNNCNV. Finally, we explore the effectiveness of each part of the KNNCNV. In addition, the performance of the above methods is quantified by precision, sensitivity, and F1-score, where precision=TP/PP , and sensitivity=TP/P , and F1-score is the harmonic mean between the precision and sensitivity. Here *TP* denotes the number of duplicate genomic positions between the declared CNVs and confirmed CNVs, and *PP* represents the total number of genomic positions in the declared CNVs, and similarly, *P* is the total number of positions in the confirmed CNVs. In terms of the real cancer datasets, the comparison of our method with peer methods is made in terms of the overlapping density score (ODS) (Yuan et al., 2020). To fairly compare our method with existing ones, their default parameters are used. Note that the performance of the KNNCNV on a third-generation sequencing sample is shown in Supplementary Table 1.

### Simulation Studies

The simulated datasets were generated by the IntSIM (Yuan et al., 2017), and two key parameters (*i.e.*, tumor purity and coverage depth) should be specified. In each simulated configuration, the tumor purity ranged from 0.2 to 0.4 in increments of 0.1, and the coverage depth belonged to the set {4x, 6x}. In addition, chromosome 21 of hg19 was selected as the reference genome. To simplify the representations, we use (*p*, *cov*) to represent the tumor purity and coverage depth, respectively. Note that each simulated configuration was repeated fifty times to reduce the randomness of the experiments, and their average performance was reported.

**FIGURE 2:**
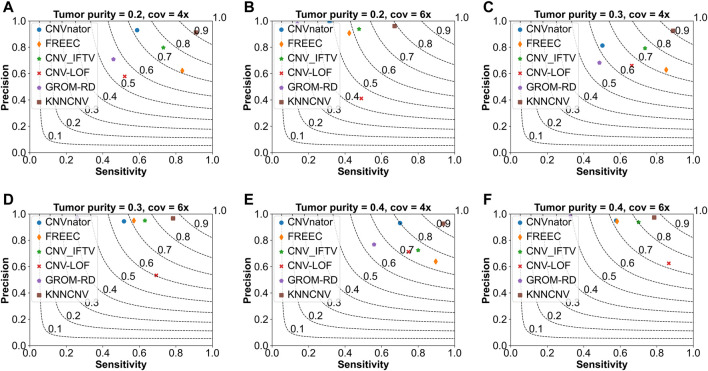
Comparison of the KNNCNV with five peer methods on the simulated datasets in terms of precision, sensitivity, and F1-score. The F1-score is shown in black dashed lines ranging from 0.1 to 0.9 with an increment of 0.1. **(A–F)** They show the performance of the above six methods on different simulated configurations, respectively.

To show the effectiveness of the KNNCNV, the comparisons of the KNNCNV with five existing methods are shown in Figure 2, and these existing methods include CNVnator (Abyzov et al., 2011), FREEC (Boeva et al., 2010; Boeva et al., 2012), CNV_IFTV (Yuan et al., 2021b), CNV-LOF (Yuan et al., 2021a), and GROM-RD (Smith et al., 2015). One can observe that the sensitivity of our method outperforms other methods except for Figure 2F. Furthermore, although the KNNCNV is not very prominent in precision and sensitivity, it achieves a surprised F1-score compared to these existing methods. More precisely, in terms of F1-score, the KNNCNV is about 14.33%, 14.28%, 13.84%, 9.07%, 12.64%, and 5.28% higher than the highest existing methods, respectively.

**FIGURE 3:**
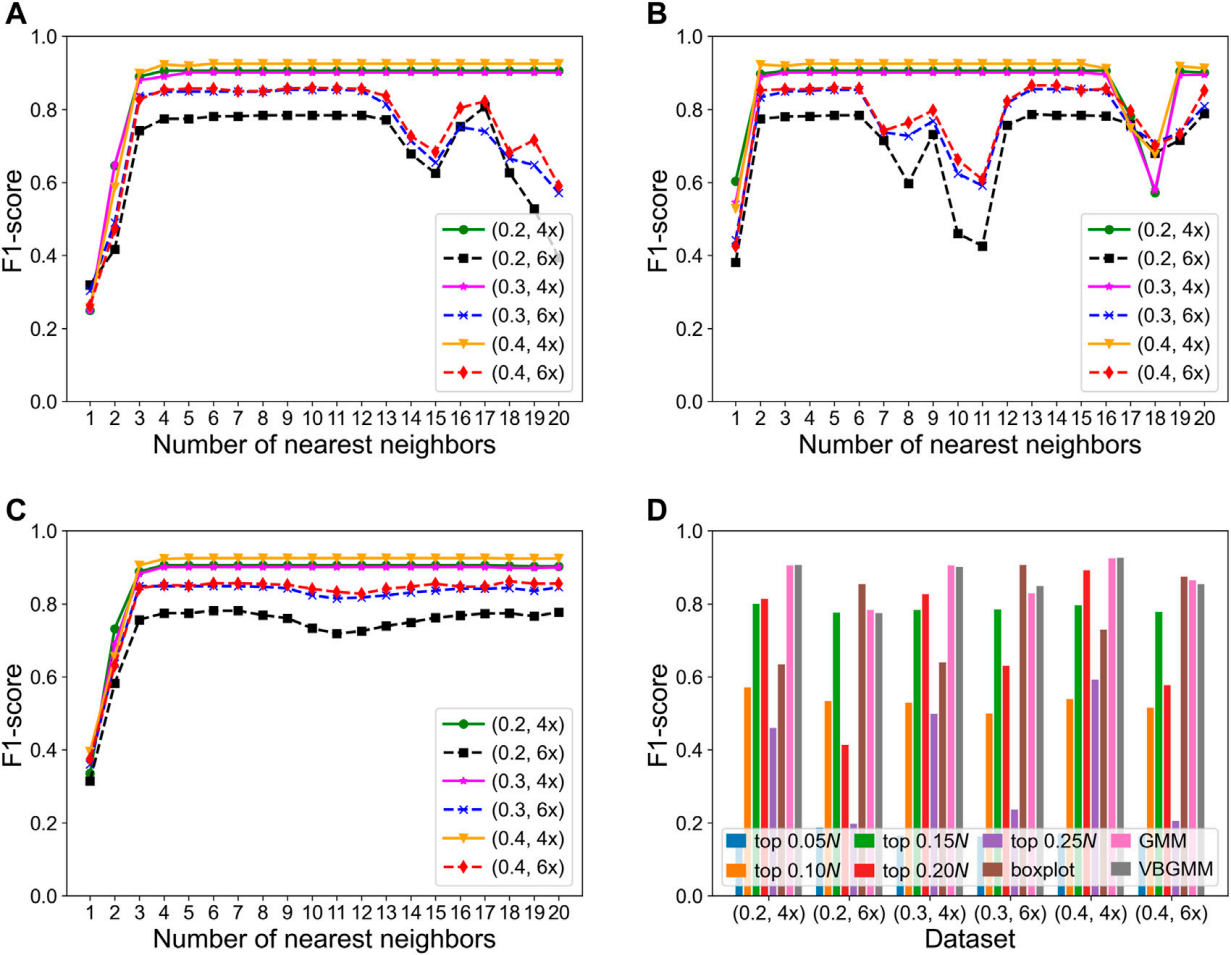
F1-score on the simulated datasets. **(A-C)** Variation of different outlier score schemes generated by the KNN with the number of nearest neighbors, where 1–20 denote 0.05*, 0.10*, 0.15*N*, … , 0.95*N*, and 1.00*N*, respectively. The different outlier score schemes are the median outlier score scheme **(A)**, the largest outlier score scheme **(B)**, and the average outlier score scheme **(C)**. **(D)** Result with different threshold selection strategies under the framework of our method, where the ‘top *n*’ denotes a simple threshold, and *n* belongs to the set {0.05*, 0.10, 0.15, 0.20*, 0.25*N*}. Specifically, after the outlier scores are ranked in descending order, the scheme treats the genome segments corresponding to the first *n* scores as CNVs. Note that 1–20 and *n* are rounded down.

**FIGURE 4:**
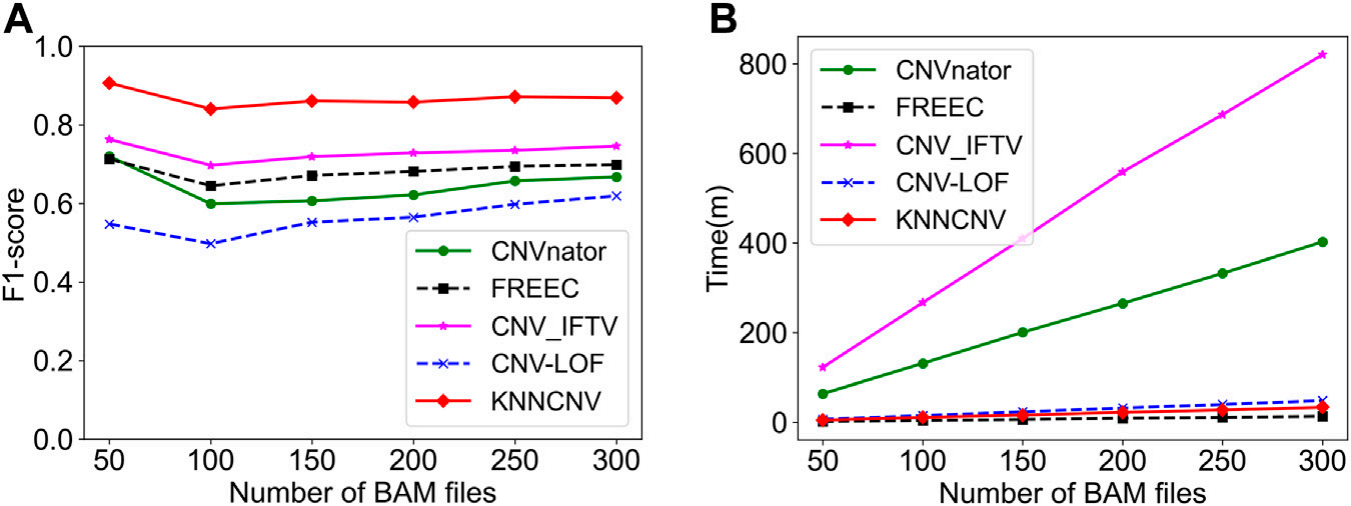
Comparison of the KNNCNV with four peer methods on the simulated datasets in terms of F1-score and computational cost. **(A)** Performance of the five methods with different the number of BAM files. **(B)** Variation of running time of the five methods with the number of BAM files.

The KNNCNV involves some hyperparameters including bin size, region size, and the number of nearest neighbors (*i.e.*, *k*). Among them, only the *k* value has been carefully researched, so Figures 3A–C show the variation of different outlier score schemes generated by the KNN with the number of nearest neighbors, respectively. The results indicate that the median outlier score scheme and the largest scheme may yield unstable performance when the *k* value is not reasonable, and the average outlier score scheme is relatively insensitive to the *k* value when it reaches a certain value. Additionally, we study the effectiveness of the VBGMM (Corduneanu and Bishop, 2001; Tzikas et al., 2008). The comparison of the VBGMM with other threshold selection strategies is shown in Figure 3D, and these threshold selection strategies consist of some simple threshold schemes, boxplot (Sim et al., 2005), and GMM (Bishop, 2007; Aggarwal, 2017). Note that the boxplot scheme treats the upper fence of the boxplot as CNVs, and its whisker is 0.75. It can be seen that the GMM and the VBGMM outperform other strategies for *cov* = 4x. Although the boxplot scheme ranks first for *cov* = 6x, the one is less stable than the GMM and the VBGMM. Furthermore, a simple threshold scheme is also desirable when a suitable threshold is found, but it is difficult to find such a threshold in real-world applications. To further discuss the complexity of the proposed method, the computational cost and performance of five methods vary with the number of BAM files, as shown in Figures 4A,B, and these five methods include CNVnator, FREEC, CNV_IFTV, CNV-LOF, and KNNCNV. The results show that the KNNCNV not only has promising performance, but also its computing overhead is acceptable.

### Application to Real Datasets

#### Analysis of Blood Samples from the 1,000 Genomes Project

The real blood samples consist of NA12878, NA12891, NA12892, NA19238, NA19239, and NA19240, where the first three samples come from the CEU trio of European ancestry and the remaining three from the YRI trio of Yoruba Nigerian ethnicity. Note that each trio includes two parents and one daughter, and the above six samples can be obtained from the 1,000 Genomes Project (http://www.1000genomes.org). Each real sequencing sample was repeated twenty times on the 21st chromosome, and their average performance was reported. The confirmed CNVs of these samples can be obtained from the database of genomic variants (http://dgv.tcag.ca/dgv/app/home), which can help us calculate some performance metrics, such as precision, sensitivity, and F1-score.

**FIGURE 5:**
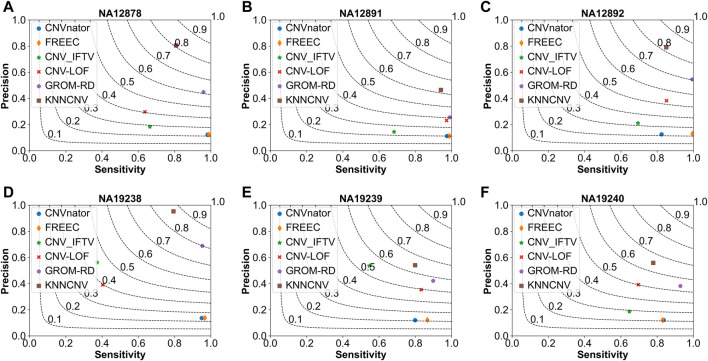
Comparison of the KNNCNV with five peer methods on the real datasets in terms of precision, sensitivity, and F1-score. The F1-score is shown in black dashed lines ranging from 0.1 to 0.9 with an increment of 0.1. **(A-F)** They show the performance of the above six methods on six real datasets, respectively.

As shown in Figure 5, we make comparisons between the KNNCNV and five peer methods on the six real datasets. It can be observed that our method achieves the best F1-score, outperforming the highest existing method by 19.57%, 21.45%, 11.50%, 6.61%, 7.17%, and 11.00%, respectively. Furthermore, the precision of our method also significantly outperforms these peer methods. Additionally, the precision of many methods is unsatisfactory compared to Figure 2 since there is a certain deviation between the simulated and real datasets. Specifically, due to the complexity of realistic cancer genomes, the simulated datasets cannot accurately reflect the variant distributions and correlations of the real datasets and do not take into account insertion and deletion errors.

**FIGURE 6:**
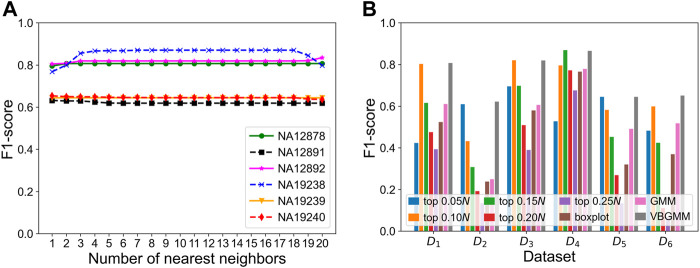
Performance of the KNNCNV in terms of F1-score on the six real datasets. **(A)** Performance of our method with different the number of nearest neighbors, where 1–20 denote 0.05*, 0.10*, 0.15*N*, … , 0.95*N*, and 1.00*N*, respectively. **(B)** Result with different threshold selection strategies under the framework of our method, where *D*_1_-*D*_6_ denote NA12878, NA12891, NA12892, NA19238, NA19239, and NA19240, respectively. Additionally, the ‘top *n*’ represents a simple threshold scheme, and *n* belongs to the set {0.05*, 0.10, 0.15, 0.20*, 0.25*N*}. Specifically, after the outlier scores are ranked in descending order, the scheme treats the genome segments corresponding to the first *n* scores as CNVs. Note that 1–20 and *n* are rounded down, and *N* is the number of genome segments.

To verify the effectiveness of each part of the KNNCNV, the hyperparameter *k*, other threshold selection strategies, different outlier score schemes generated by KNN, and other detector schemes are discussed. The variation of the KNNCNV with the number of nearest neighbors is shown in Figure 6A. The result illustrates that the performance of the KNNCNV is less sensitive to the *k* value when it reaches a certain value. Furthermore, our method has slight fluctuations in performance for *k* = 1.00*N*, as some local CNVs may be ignored. Figure 6B shows the result with different threshold selection strategies under the framework of our method, and these threshold selection strategies contain some simple threshold schemes, boxplot, GMM, and VBGMM. One can observe that in addition to a single simple threshold scheme, the VBGMM is significantly better than other threshold selection strategies. Additionally, although the simple threshold scheme (*i.e.*, top *n*) is promising when a suitable *n* value is found, such as ‘top 0.10*N*’ on NA12878 and ‘top 0.05*N*’ on NA19239, it is a challenge to determine the *n* value in real-world applications. To prove the effectiveness of the KNN detector, the comparison of the KNN with the LOF (Breunig et al., 2000) and the IF (Liu et al., 2012) detector is shown in Table 1. ‘Detector + VBGMM’ denotes that Detector calculates the outlier scores for all genome segments, and these scores are transformed into binary labels by the VBGMM. Note that the input of Detector is the RD values for all genome segments (*i.e.*, *R*), and ^*^ represents the KNNCNV. Here the highest value in each column is highlighted. The result indicates that KNN outperforms LOF and IF except for NA12891 and ranks first in the average performance among the six real datasets.

**TABLE 1:** F1-score on six real blood datasets.

Methods	NA12878	NA12891	NA12892	NA19238	NA19239	NA19240	Average
IF + VBGMM	0.3790	0.1553	0.4247	0.5455	0.3764	0.3015	0.3637
LOF + VBGMM	0.6424	**0.6453**	0.6813	0.6906	0.6290	0.5968	0.6476
KNN + VBGMM^*^	**0.8068**	0.6325	**0.8194**	**0.8658**	**0.6449**	**0.6444**	**0.7356**

#### Analysis of Cancer Samples from the European Genome-Phenome Archive

The cancer samples involve a lung cancer sample (*i.e.*, EGAD00001000144_LC) and two ovarian cancer samples (*i.e.*, EGAR00001004802_2053_1 and EGAR00001004836_2561_1), and they can be obtained from the European Genome-Phenome Archive (https://ega-archive.org/). These samples are genome-wide samples (22 autosome chromosomes) and have no confirmed CNVs (*i.e.*, ground truth). Thus, the performance of methods cannot be quantified by the precision, sensitivity, and F1-score. As a remedy, the ODS is adopted to quantify the performance of methods, and the ODS for the *j*th method can be formulated as Eq. 13.

$$ODS\left(j\right)=m{\left(j\right)}_{cnv}\cdot m^{\prime }{\left(j\right)}_{cnv},$$ (13)

where the definitions of $m{\left(j\right)}_{cnv}$ and $m^{\prime }{\left(j\right)}_{cnv}$ refer to the prior work (Yuan et al., 2020). The comparison of the KNNCNV with peer methods on the three genome-wide samples (22 autosome chromosomes) is shown in Table 2, and these peer methods consist of CNVnator, FREEC, CNV_IFTV, and CNV-LOF. Here the highest value in each row is shown in bold. The result illustrates that in samples EGAD00001000144_LC and EGAR00001004836_2561_1, the KNNCNV outperforms peer methods and ranks second in the remaining sample. In addition, our method achieves the highest average ODS among the three genome-wide samples.

**TABLE 2:** ODS on three genome-wide samples (22 autosome chromosomes).

Sample	CNVnator	FREEC	CNV_IFTV	CNV-LOF	KNNCNV
EGAD00001000144_LC	0.0062	0.0026	0.0212	0.1452	**1.1204**
EGAR00001004802_2053_1	0.1176	0.1488	3.4559	**10.5594**	8.6538
EGAR00001004836_2561_1	0.6182	2.4948	0.9263	2.8583	**5.6250**
Average	0.2473	0.8821	1.4678	4.5210	**5.1331**

## Discussion

This paper proposes a new method used for whole genome sequencing, called KNNCNV, which can detect CNVs using NGS data. The KNNCNV first calculates the outlier score for any genome segment based solely on its first *k* nearest-neighbor distances. Specifically, the average value of these distances is considered as the outlier score for the genome segments. Finally, based on the VBGMM, these scores for all genome segments are converted into a succession of binary labels to indicate which genome segments are CNVs. Note that the outlier score calculation schemes for KNNCNV and CNV-LOF (Yuan et al., 2021a) are all based on the first *k* nearest-neighbor distances between a genome segment and the remaining ones. The difference between these two types of scores is that the KNNCNV treats solely the average value of the first *k* nearest-neighbor distances as its scores, while the scores of CNV-LOF require the further calculation of reachability distance, local reachability density, and local outlier factor. Thus, in contrast to CNV-LOF, KNNCNV is not only simpler but also has less computing overhead. Compared to the existing methods, the KNNCNV has two key features: 1) the outlier score for any genome segment can be obtained by the average outlier score scheme, which is not only easy to extend to other data types but also improves the power of detection CNVs, especially the local CNVs that are likely to be masked by their surrounding regions; 2) the posterior distribution of these scores is approximated by the VBGMM, which can obtain a series of binary labels without a pre-determined threshold.

We conduct experiments on simulated and real datasets to show the effectiveness of the KNNCNV. The comparisons of our method with peer methods are made, and the results show that the KNNCNV achieves encouraging performance in terms of F1-score. In addition, we verify the effectiveness of each part of the KNNCNV. The results indicate that the VBGMM is an effective threshold selection strategy, and the KNN is a simple and effective detector. Therefore, the KNNCNV might become a promising tool for the detection of CNVs.

As for the potential disadvantages of our method, when calculating the outlier scores for all genome segments, there is a natural assumption that the *k*-nearest neighbor distance of outliers (*i.e.*, CNVs) is much larger than that of normal points. In other words, it assumes that the CNVs regions only account for a small fraction of the whole genome. However, the CNVs regions may cover a large fraction of the whole genome in some cancers, so the KNNCNV may not detect CNVs accurately in that case. In future work, we would be dedicated to solving the case that the CNVs regions account for a large proportion of the whole genome.

## Data Availability

The original contributions presented in the study are included in the article/Supplementary Material, further inquiries can be directed to the corresponding author.
