# Supplementary material for: KNNCNV: A K-Nearest Neighbor Based Method for Detection of Copy Number Variations Using NGS Data
Source: Front Cell Dev Biol. 2021 Dec 22;9:796249. doi: 10.3389/fcell.2021.796249 (PMC8728060; doi:10.3389/fcell.2021.796249)
Supplement: Supplementary file 1 [file DataSheet1.PDF]

Supplementary Material

1 Analysis of a Third-generation Sequencing Sample

The third-generation sequencing sample is NA12878 that is a genome-wide sample (22 autosome chromosomes) and from the CEPH1463 human genome reference standard on the Oxford Nanopore MinION using 1D ligation kits (450 bp/s) using R9.4 chemistry (FLO-MIN106) (Jain et al., 2018). Also, the sample can be download from the http://s3.amazonaws.com/nanopore-human-wgs/rel3-nanopore-wgs-4244727060-FAB23716.fastq.gz.sorted.bam. Due to the absence of the confirmed CNVs (*i.e.*, ground truth), the overlapping density score (ODS) (Yuan et al., 2020) is adopted to quantify the performance of methods, and these methods include CNVnator (Abyzov et al., 2011), FREEC (Boeva et al., 2010; Boeva et al., 2012), CNV_IFTV (Yuan et al., 2021b), CNV-LOF (Yuan et al., 2021a), and KNNCNV. The result of the above methods is shown in **Supplementary Table 1**, and the highest value in each row is shown in bold. The result indicates that the CNV-LOF outperforms other methods, followed by the KNNCNV.

Supplementary Table 1 | ODS on a third-generation sequencing sample

Sample	CNVnator	FREEC	CNV_IFTV	CNV-LOF	KNNCNV
NA12878	0.1378	1.0016	0.044	**1.6461**	1.4167

2 Reference

Abyzov, A., Urban, A.E., Snyder, M., and Gerstein, M. (2011). CNVnator: an approach to discover, genotype, and characterize typical and atypical CNVs from family and population genome sequencing. *Genome Research* 21(6)**,** 974-984. doi: 10.1101/gr.114876.110.

Boeva, V., Popova, T., Bleakley, K., Chiche, P., Cappo, J., Schleiermacher, G., et al. (2012). Control-FREEC: a tool for assessing copy number and allelic content using next-generation sequencing data. *Bioinformatics* 28(3)**,** 423-425. doi: 10.1093/bioinformatics/btr670.

Boeva, V., Zinovyev, A., Bleakley, K., Vert, J.-P., Janoueix-Lerosey, I., Delattre, O., et al. (2010). Control-free calling of copy number alterations in deep-sequencing data using GC-content normalization. *Bioinformatics* 27(2)**,** 268-269. doi: 10.1093/bioinformatics/btq635.

Jain, M., Koren, S., Miga, K.H., Quick, J., Rand, A.C., Sasani, T.A., et al. (2018). Nanopore sequencing and assembly of a human genome with ultra-long reads. *Nature Biotechnology* 36(4)**,** 338-345. doi: 10.1038/nbt.4060.

Yuan, X., Bai, J., Zhang, J., Yang, L., Duan, J., Li, Y., et al. (2020). CONDEL: detecting copy number variation and genotyping deletion Zygosity from single tumor samples using sequence data. *IEEE/ACM Transactions on Computational Biology and Bioinformatics* 17(4)**,** 1141-1153. doi: 10.1109/TCBB.2018.2883333.

Yuan, X., Li, J., Bai, J., and Xi, J. (2021a). A local outlier factor-based detection of copy number variations from NGS data. *IEEE/ACM Transactions on Computational Biology and Bioinformatics* 18(5)**,** 1811-1820. doi: 10.1109/TCBB.2019.2961886.

Yuan, X., Yu, J., Xi, J., Yang, L., Shang, J., Li, Z., et al. (2021b). CNV_IFTV: an isolation forest and total variation-based detection of CNVs from short-read sequencing data. *IEEE/ACM Transactions on Computational Biology and Bioinformatics* 18(2)**,** 539-549. doi: 10.1109/TCBB.2019.2920889.
